# Fulminant Coxsackievirus Myocarditis in an Immunocompetent Adult: A Case Report and Literature Analysis

**DOI:** 10.7759/cureus.25787

**Published:** 2022-06-09

**Authors:** Ashish Jain, Rahul P Rane, Maha Mumtaz, Asfand Y Butt, Mahmoud Abdelsalam, Saba Waseem

**Affiliations:** 1 Internal Medicine, Conemaugh Memorial Medical Center, Johnstown, USA

**Keywords:** enterovirus, fulminant cardiomyopathy, coxsackie virus, nonischemic cardiomyopathy, fulminant myocarditis

## Abstract

Myocarditis is an inflammatory condition that impacts cardiac myocytes and is caused mostly by viruses. It can manifest as chest pain, dyspnea, palpitations, fatigue, syncope, shortness of breath, and in severe cases frank cardiogenic shock. It accounts for around 10 percent of all sudden cardiac deaths in young adults, who are described as being in their early thirties. Inflammatory cardiomyopathy resulting from acute myocarditis may also appear as new-onset heart failure (HF), delaying the diagnosis and treatment of these patients. It is crucial to recognize the sensitivity of symptom onset, especially in young individuals; mildly elevated troponin levels that are inconsistent with the severity of left ventricular ejection fraction (LVEF) impairment and associated left ventricular dilatation strongly suggest inflammatory cardiomyopathy rather than acute myocarditis. The current treatment for myocarditis is primarily supportive, with an emphasis on the management of heart failure and arrhythmias in accordance with clinical practice guidelines. In this case report, we describe a male in his early forties who presented with abrupt onset exertional shortness of breath and chest discomfort. His cardiac catheterization did not show evidence of coronary artery disease; however, an echocardiogram revealed new-onset heart failure with reduced ejection fraction, the diagnosis of coxsackievirus myocarditis was made based on his clinical presentation, and a positive coxsackievirus immunoassay.

## Introduction

Inflammatory cardiomyopathy refers to myocarditis in conjunction with cardiac dysfunction and myocardial remodeling. This illness is associated with poor outcomes when it is associated with left ventricular dysfunction, heart failure, or arrhythmia, despite progress in our understanding of the disorder's pathophysiology due to extensive research, fulminant myocarditis is one of the leading causes of cardiogenic shock in young adults [1]. To reduce mortality and the need for heart transplantation within this patient population, early identification and individualized treatment options are required. In this literature review and case report, we attempt to highlight the pathophysiology, presentation, diagnosis, management, and prognosis of inflammatory cardiomyopathy associated with coxsackievirus myocarditis.

## Case presentation

**Figure 1:**
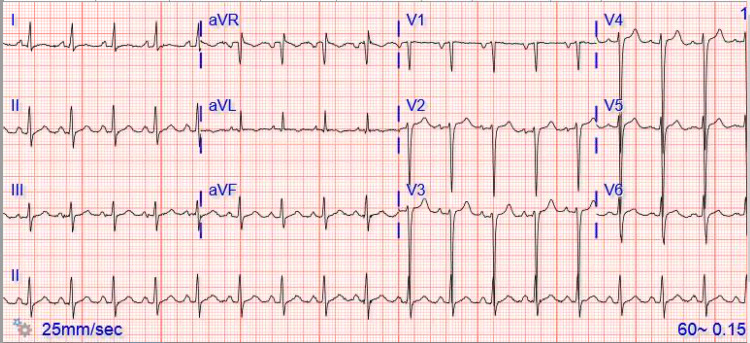
EKG EKG showed normal axis sinus tachycardia, poor R wave progression, and no substantial ST segment or T wave changes.

We present a case of a male in his early forties with a past medical history of alcohol use disorder who presented with intermittent complaints of progressively worsening exertional shortness of breath and 5 out of 10 left-sided and non-radiating chest discomfort over the last three weeks. Sinus tachycardia with a normal axis and poor R wave progression with no substantial ST segment and T wave alterations were detected on the EKG (Figure 1).

**Figure 2:**
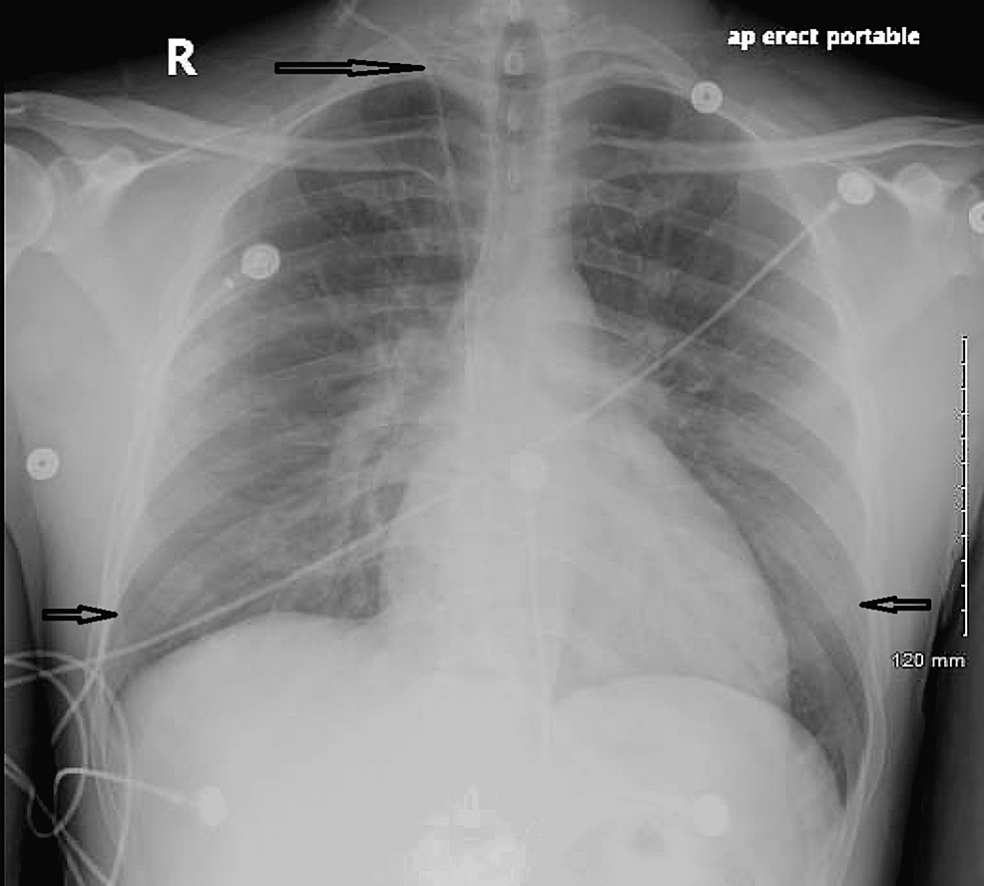
X-ray chest in a single view The central line of the right internal jugular vein (IJV) as highlighted by an arrow is visible, with the tip protruding into the predicted location of the mid- superior vena cava (SVC).  There is no evidence of pleural effusion or pneumothorax. Bilaterally, ill-defined patchy mild interstitial alveolar opacities are visible as highlighted by arrows.

Chest X-ray revealed mild cardiomegaly, new minor bilateral perihilar pulmonary vascular congestion, and a small right pleural effusion, all of which are novel findings when compared to the previous chest film and were most consistent with pulmonary edema or congestive heart failure (CHF) (Figure 2).

The laboratory findings were significant for marginally elevated high-sensitivity troponin levels and brain natriuretic peptide (BNP) levels of 555 pg/ml. He underwent echocardiography, which revealed an LVEF of 25-30% and mild to severe mitral regurgitation (Video 1).

**Video 1:** Video representation of patients echocardiogram. Parasternal Long Axis View (PLAX), Parasternal Short Axis View (PSAX), Apical Four Chamber View (A4C), and Apical Two chamber View (A2C) Indicating Reduced Left Ventricular function. Increased E Point Septal Separation (EPSS) indicating towards reduced Left Ventricular Function

Following the discovery of left ventricular dysfunction, he was referred for cardiac catheterization, which ruled out ischemic cardiomyopathy, however, the procedure was complicated by sudden cardiogenic shock, requiring pressor support including dopamine and later milrinone and norepinephrine. Initially, it was assumed that the patient had dilated cardiomyopathy secondary to alcohol use, but a viral panel revealed coxsackievirus (immunoglobulin{Ig}M and IgG) positivity, indicating fulminant myocarditis leading to cardiogenic shock. He was discharged with guideline-directed medical therapy including a wearable defibrillator, and cardiac rehabilitation. He was followed as an outpatient by primary care and cardiology; additionally, the patient continued to complain of exertional shortness of breath, and because he met the criteria for an implantable cardioverter-defibrillator (ICD), he was scheduled for the procedure in four months after the diagnosis. The patient then underwent ICD placement and follow-up echocardiography that revealed a left ventricular ejection fraction of 30-35% as compared to the prior scans.

## Discussion

Pathogenesis

As the name implies, myocarditis is an inflammatory illness involving cardiac myocytes and is primarily caused by viruses. However, several bacteria, protozoa, and fungus have been implicated in the disease's causation. Coxsackievirus is an enterovirus that has a long history of being associated with acute myocarditis and inflammatory cardiomyopathy. Coxsackievirus binds to a transmembrane receptor on cardiac myocytes coxsackievirus and adenovirus receptor (CAR) [2]. It directly injures the myocardium and disrupts the cytoskeletal structure, triggering an uncontrolled immunological response that can continue even after the virus is eradicated. Coxsackievirus is a cytolytic virus; it replicates within the host cell and then lyses the cell, causing myocarditis. Chronic coxsackievirus infection of cardiac myocytes is associated with impaired cardiac ventricular function, which results in poor clinical outcomes and increased mortality; however, around half of patients recover fully without substantial clinical consequences [3]. Both heterozygous and homozygous carriers of the CCR5Δ32 gene deletion have been reported to clear enteroviruses spontaneously, in comparison to individuals bearing the wild-type CCR5, implying a genetic basis for disease development and outcome [4].

Presentation 

Acute myocarditis can appear with chest discomfort, dyspnea, palpitations, tiredness, syncope, shortness of breath, and frank cardiogenic shock. It accounts for around 10% of all-cause mortality from sudden cardiac death in young adults, defined as those in their early thirties [3]. Flu-like symptoms, gastrointestinal disturbances, and fever are prodromal signs that occur in the weeks preceding the acute phase of myocarditis and occur in approximately 80% of patients with acute myocarditis [5]. The type of presentation has been shown to be predictive of disease outcome. For example, when patients with features of complicated myocarditis had a left ventricular ejection fraction (LVEF) of 50% on initial echocardiography, hemodynamic instability, or sustained tachycardia were compared to patients with features of uncomplicated myocarditis, the risk of requiring a heart transplant or cardiac death was 14.7 percent at five years [5]. Inflammatory cardiomyopathy owing to acute myocarditis may also manifest with new-onset heart failure (HF), leading to delayed diagnosis and care for these individuals. It is critical to associate the sensitive nature of symptom onset, particularly in young individuals; mildly elevated troponin levels that are not consistent with the severity of LVEF impairment with associated left ventricular dilatation are highly suggestive of inflammatory cardiomyopathy rather than acute myocarditis. Unfortunately, there are few established and consistent clinical markers for predicting the prognosis of individuals with inflammatory cardiomyopathy.

Late gadolinium enhancement (LGE) distribution pattern assessment on MRI has been shown to improve risk stratification in patients with preserved LVEF, and histological patterns such as giant-cell myocarditis and eosinophilic myocarditis are independently associated with increased mortality and can be used as a prognostic indicator in patients with fulminant myocarditis who present with features of cardiogenic shock [6,7]. Inflammatory cardiomyopathy owing to acute myocarditis may also manifest with new-onset HF leading to delayed diagnosis and care for these individuals. It is critical to associate the sensitive nature of symptom onset, particularly in young individuals; mildly elevated troponin levels that are not consistent with the severity of LVEF impairment with associated left ventricular dilatation are highly suggestive of inflammatory cardiomyopathy rather than acute myocarditis [3]. Unfortunately, there are few established and consistent clinical markers for predicting the prognosis of individuals with inflammatory cardiomyopathy.

Diagnosis****

Endomyocardial biopsy (EMB) plus histological and immunohistochemical examination continues to be the gold standard for diagnosing myocarditis. The Dallas Criteria for Active Myocarditis uses histopathological analysis to define active myocarditis as inflammatory infiltrates of the myocardial tissue that is typically lymphocytic and macrophagic but can also be neutrophilic or eosinophilic, with necrosis and degeneration of neighboring myocardial cells that are not characteristic of damage caused by ischemia associated with coronary artery disease. Interobserver heterogeneity in specimen interpretation and the inability to detect noncellular inflammation continues to be the Dallas Criteria's primary limitations, producing diagnostic relevance in just 10-20% of patients [8,9]. Immunohistochemical staining using monoclonal and polyclonal antibodies improves diagnostic and prognostic accuracy, which can be further enhanced by molecular identification of viral genomic sequences in diseased myocardium [10]. Active myocarditis is defined by the World Health Organization (WHO) as focal or diffuse mononuclear infiltrates, defined as lymphocytes and macrophages with a density of >14 cells/mm^2^, and elevated expression of human leukocyte antigen (HLA) class II molecules [11]. When conducted early in the illness course, endomyocardial biopsy provided the greatest diagnostic and prognostic information. However, it is restricted for a variety of reasons, the most significant of which is an invasive intrathoracic treatment.

According to the 2007 AHA/ACC/ESC consensus statement on the role of endomyocardial biopsy (EMB), Class I recommendations for early EMB are reserved for patients with fulminant cardiomyopathy which is unresponsive to conventional therapy or unexplained cardiomyopathy with associated conduction diseases or ventricular arrhythmias [12]. However, because this recommendation was based on the Dallas Criteria, which utilizes histopathological analysis, the 2013 European society of cardiology position statement on the role of EMB calls for broader use of EMB in conjunction with immunohistochemistry and viral genomic studies in the assessment of patients with suspected myocarditis [13]. According to the European society of cardiology (ESC) guidelines, cardiac magnetic resonance imaging (CMR) remains the noninvasive gold standard technique for detecting myocarditis. The inclusion of T2-weighted CMR to the established Lake Louise criteria resulted in an increase in diagnostic power due to the enhanced sensitivity. CMR accuracy varies with the amount of necrosis and clinical presentation in patients with biopsy-proven acute myocarditis. CMR has a high sensitivity for infarction-like presentations, a low sensitivity for cardiomyopathy-like presentations, and a very low sensitivity for patients presenting with arrhythmia; additionally, the etiology and type of myocarditis cannot be determined in routine clinical practice using CMR [9,13].

Management 

Currently, treatment for myocarditis is mostly supportive, with an emphasis on the guideline-directed treatment of heart failure and arrhythmias [13]. To our knowledge, no pathogen-directed or antiviral medications are authorized for patients with viral myocarditis. Management with acyclovir, valaciclovir, or ganciclovir, for herpes virus infection, may be attempted, although their effectiveness in patients with myocarditis has not been studied directly. Mechanical assistance may be required for patients who present with hemodynamic instability, either as a link to transplantation or remission [9]. Parenteral inotropes and brief mechanical circulatory support (MCS) devices are frequently required in patients with cardiogenic shock caused by fulminant myocarditis. MCS can be utilized regardless of when immunosuppressive medication is initiated. Various MCS devices are effective for temporary hemodynamic stabilization and can act as a bridge to transplantation in patients with fulminant myocarditis, including intra-aortic balloon pumps (IABPs), veno-arterial extracorporeal membrane oxygenation (V-A ECMO), percutaneous ventricular assist devices (VAD), and Impella micro axial flow catheters. These devices behave differently, most notably in terms of afterload modification. The effect of these devices on afterload modulation may be particularly relevant in patients with fulminant myocarditis since an increase in peripheral resistance could further promote cardiac inflammatory responses via an increase in ventricular wall stress [3]. Individuals with ventricular tachycardia or ventricular fibrillation during the acute phase of myocarditis have a significantly increased risk of recurrence of persistent ventricular arrhythmias and may benefit from an ICD [14].

## Conclusions

Viral myocarditis is an uncommon condition but has a significant fatality rate. Early diagnosis and therapy initiation are crucial for survival. Endomyocardial biopsy (EMB) plus histological and immunohistochemical examination continues to be the gold standard for diagnosing myocarditis. Treatment for myocarditis is mostly supportive, with an emphasis on the guideline-directed treatment of heart failure and arrhythmias. As a link to transplantation or remission, patients who present with hemodynamic instability may require mechanical support.
